# A time with e-Natureza (e-Nature): a model of nature-based health interventions as a complex adaptive system

**DOI:** 10.3389/fpsyg.2023.1226197

**Published:** 2023-08-22

**Authors:** Eliseth Ribeiro Leão, Erika Hingst-Zaher, Roberta Maria Savieto, Karina Pavão Patricio, Leticia Bernardes de Oliveira, Giulia Catissi, Luciano Moreira Lima, Gustavo Benvenutti Borba, Sabrina Bortolossi Bomfim, Floriana Bertini de Abreu

**Affiliations:** ^1^Albert Einstein Education and Research Center, Hospital Israelita Albert Einstein, São Paulo, Brazil; ^2^Museu Biológico, Instituto Butantan, São Paulo, Brazil; ^3^Medical School of Botucatu, UNESP, São Paulo, Brazil; ^4^Pediatric and Neonatology Unit, Hospital Israelita Albert Einstein, São Paulo, Brazil; ^5^Albert Einstein Israelita Faculty of Health Sciences, Hospital Israelita Albert Einstein, São Paulo, Brazil; ^6^Department of Electronics, Graduate School on Biomedical Engineering, Federal University of Technology–Paraná, Curitiba, Brazil; ^7^Diagnostic Services, Hospital Israelita Albert Einstein, São Paulo, Brazil

**Keywords:** nature-based interventions, health promotion, models, complex adaptative systems, forest bathing, nature relatedness, nature conservation

## Abstract

Discussions surrounding the positive impacts of nature on human health and strategies to enhance our connection with the natural world have been ongoing. However, a limited number of theoretical models are available to guide research and practice in this area. Therefore, there is a pressing need for a systematic framework that outlines clear steps for conducting research implementing nature-based interventions. In this study, we investigate the relationship between health and nature through the lens of Complex Adaptive Systems. This approach involves examining the dynamic interactions between multiple interconnected elements to understand the complex emergent behaviors that arise from such relationships. Our model is designed to support nature-based interventions, considering the essential interdependence between humans and nature. This perspective aims to improve both human health and biodiversity conservation in a mutually beneficial manner. The underlying interactions that drive nature-based health interventions are thoroughly explored, leading us to propose a novel intervention model named “A time with e-Natureza” (e-Nature). This model encompasses four types of experiences, drawing from scientific literature and insights from authors engaged in an interdisciplinary research group: (1) Aesthetic and emotional experience; (2) Multisensory integration experience; (3) Knowledge experience; and (4) Engagement experience. Each experience within the model targets affective, cognitive, and behavioral aspects, with a specific focus on fostering a deeper connection with nature. Distinct activities are incorporated within each experience to promote successful outcomes. The model is grounded in existing theories that address the human-nature relationship and is informed by Nursing theories that support health promotion interventions. By presenting this new model, our aim is to contribute to the effective implementation of nature-based interventions that not only enhance human well-being but also support the conservation of nature. This integrated approach recognizes the mutual benefits of human-nature interaction and offers valuable insights for future research and practical applications in the fields of nature and health.

## 1. Introduction

The world has been undergoing rapid and threatening changes for humanity. Climate change lies at the heart of various current problems that are likely to increase in number and severity if the relationship between human beings does not return to positive levels of coexistence and interdependence that have permeated their existence on this planet (Pörtner et al., 2022).

The recent report of the Intergovernmental Panel on Climate Change (IPCC) have highlighted the unequivocal role of human activities in driving the process of climate change, leading to significant changes in atmospheric, oceanic, and terrestrial surface warming (Lee et al., 2023). This has significantly affected the foundations of human health and well-being (Romanello et al., 2022). Catastrophic floods and storms have forced over 20 million people to leave their homes each year. Forest fires are burning larger areas than ever before in many regions, and higher temperatures are also facilitating the spread of vector-borne diseases, increasing the likelihood of emerging and re-emerging infectious diseases. Climate change is affecting entire species, ecosystems, and the most vulnerable people (Lee et al., 2023).

Considering the imperative for humanity to pursue a more sustainable path, the United Nations (UN) launched the 2030 Agenda in 2015. This comprehensive action plan encompasses people, the planet, and prosperity, aiming to foster universal peace, heightened freedom, and collaborative partnerships, and guided by the triple bottom line of sustainable development: economic, social, and environmental factors (United Nations, 2015).

Since 2008, there has been a significant shift in global population distribution, with most people now residing in urban areas (United Nations, 2019). This urbanization trend has raised concerns about reduced contact with nature and subsequently weaker connections to the natural world (Soga and Gaston, 2016). While it is important to note that living in urban areas does not necessarily imply a weaker connection with nature, but the limited exposure to natural environments in cities increases the risk of it happening. Pyle (1993) described as the “extinction of experience,” 30 years ago. This refers to the decline in opportunities for engaging with and accessing green and blue spaces within urban settings, leading to a diminished connection with nature.

Analyzing data from 14,745 adults in European countries, researchers found that the British have the lowest connection with nature, despite a high number of studies in the UK highlighting the implications for both people and biodiversity (Richardson et al., 2022).

Bearing in mind that what is good for humans is not always good for nature, urbanization has not only driven people away from natural areas but has also affected biological communities (Dornelas et al., 2014) and reduced biodiversity in various habitats (Dirzo et al., 2014), creating an imbalance that can affect human health. A meta-analysis of 61 experimental and observational studies found a strong overall negative correlation between biodiversity and disease risk (Civitello et al., 2015).

In addition, since the post-World War II era, technological change and the emergence of indoor and virtual recreational options have played a role in distancing people from natural environments. Examples of these recreational options include television in the 1950s, videogames in the 1970s, and the advent of the internet, mobile computers, and smartphones from the 2000s onwards. Some data also demonstrate that nature is significantly less present in popular culture today than in the first half of the 20th century, with a steady decline after the 1950s as well (Kesebir and Kesebir, 2017). Technology potentially reduces our connection to nature, at a cost to the well-being of people and the environment that sustains us, and there is growing concern about the potential for smartphone addiction. However, a greater connection with nature can provide a respite from smartphone use, yet it should not simply be framed as an antidote (Richardson et al., 2018).

Considering this context, scientists around the world have been discussing this issue in search of ways to improve human connection with nature, as well as guiding models of thought, action, and behavior towards a more positive human-nature relationship (Lumber et al., 2017; Richardson and Butler, 2022).

Indeed, over the past 50 years, scientists have proposed four different conservation strategies (Mace, 2014): “Nature for itself,” in which conservation actions protect nature; “Nature despite people,” in which conservation actions restore degraded environments; “Nature for people,” which focuses on the importance of nature for human well-being, health, and the economy (as an ecosystem service); and “People and nature.” It is argued that focusing on the dual relationship, which is beneficial for both, can result in human-nature in a two-way path that can improve sustainability, but has been neglected in health and conservation policies (Barragan-Jason et al., 2023). One reason for this may be related to the lack of dialogue between these two areas of knowledge in effective collaborative projects. Additionally, there are still difficulties in systematizing strategies from the perspective of “People and Nature.”

With this in mind, in 2020, our interdisciplinary research group at the Hospital Israelita Albert Einstein in Brazil, focused on nature connection, health, and well-being, launched a series of initiatives. These included communication and awareness-raising strategies targeting the general population, educational programs designed for health and natural sciences professionals, programs for students, and a project called “Um tempo com e-Natureza” (“A time with e-Nature”) aimed at evaluating the impact of nature-based interventions on health and well-being.

The complexity theory that we adopt as a backdrop for supporting complex adaptive systems seeks to explain the complex behavior that emerges from the non-linear dynamics of systems (Chaffee and McNeill, 2007). A system is comprised of interconnected components, but simply having these components does not make it “complex.” A complex system exhibits dynamic behavior, with its actions and patterns evolving over time. These emergent behavioral patterns arise from the interactions among the system’s components within a larger whole, and cannot be reduced to the functions of individual elements within the system. Complex systems cannot be completely understood, controlled, or predicted (Meadows, 2008).

Evaluation of public health interventions from complex systems has been carried out and still lacks methodological development (McGill et al., 2021). On the other hand, natural ecosystems are complex adaptive systems that exhibit multiple alternative states and can be changed mainly in the face of the current climate crisis (Solé and Levin, 2022). Both nature and health are complex systems composed of interconnected elements. These systems exhibit dynamic and nonlinear interactions among their components, influencing each other. Understanding this complexity helps develop more effective approaches to promoting human health and nature conservation.

Considering these two systems, some aspects have been emphasized in the literature that contribute to the beneficial effects of nature for human health and well-being: the aesthetic appreciation and the apprehension of nature through the senses. Other aspects relate more directly to conservation. Integrating these experiences can be useful in a multi-component model. In view of this, the proposed model is based on four pillars: (1) Aesthetic and emotional experience; (2) Multisensory integration experience; (3) Knowledge experience; and (4) Engagement experience.

We understand that these pillars, when integrated with equal importance attributed to each, can meet the needs and benefit both humans and nature alike. The rationale for the development and the foundation of each pillar are presented in this article.

### 1.1. Health and conservation of biodiversity: a two-way road

Complex Adaptive Systems (CAS) are systems composed of multiple interconnected elements that exhibit complex emergent behaviors resulting from dynamic interactions among their components. These systems are characterized by their ability to adapt and evolve in response to changes in the environment or internal conditions. Any system can be understood as an intricate network of relationships and interactions, where the whole is more than the mere sum of its parts. Any change in any part, even in a single element, can generate reactions and modifications in the interconnected elements and in the surrounding environment. Therefore, the system is a dynamic organism that constantly adapts to internal and external changes. However, by making many small-scale changes and selecting those that produce the desired effects, individuals and groups can bring about improvements to the system (Morin, 2010). Health has been considered a CAS. The Meikirch Model of Health advocates that: “*Health is a state of well-being emerging from favorable interactions between individuals’ potentials, life demands and social and environmental determinants”* (Bircher and Kuruvilla, 2014).

Nature is often seen as a CAS because it is composed of multiple interconnected elements that adapt and evolve in response to changes in the environment. Biodiversity is an example of an emergent property of a CAS in nature. Biodiversity results from the dynamic interaction between different species, and changes in one species can have significant effects on other species and the ecosystem as a whole (Levin, 1998). It’s important to note that although humans are part of this complex system, they are also agents. The elements (or agents) that make up complex adaptive systems are independent but interconnected to other agents. As agents, their reactions, often unpredictable and underestimated, influence the entire system since interconnection is present (Plsek and Greenhalgh, 2001). Thus, both health and biodiversity are evolving and need to be considered in interventions that seek to articulate these two universes.

Complex adaptive systems is an approach adopted as the background of our rational model. Our rationale is that different types of experiences and related activities integrated, considering two complex universes, add to this double objective that can be considered a single one if we consider the concept of One Health of the World Health Organization: “*is an integrated, unifying approach that aims to sustainably balance and optimize the health of people, animals, and ecosystems. It recognizes the health of humans, domestic and wild animals, plants, and the wider environment (including ecosystems) are closely linked and interdependent*” (Adisasmito et al., 2022).

### 1.2. Nature connection – concept

The concept of connection with nature has been interpreted in various ways. Schultz defined it as “the extent to which an individual includes nature within his/her cognitive representation of self.” Its construction consists of three components: (1) Cognitive – the core of nature connection and refers to how someone feels integrated with nature; (2) Affective – the individual’s sense of care for nature; and (3) Behavioral – the individual’s commitment to protecting the natural environment (Schultz, 2002).

On the other hand, Mayer and Frantz (2004) approached connectedness from a different perspective, defining it as an individual’s affective and experiential connection to the natural world, rather than a cognitive one. Other researchers (Perrin and Benassi, 2009) proposed that connectedness with nature encompasses an individual’s beliefs and attitudes about their connection to nature, extending beyond mere affective connection.

From these concepts Geng et al. (2015) presented the concept of connection with nature, to bring together all these aspects as “the feelings of an individual not only emotionally, but also cognitively in relation to connections with nature and belonging “. It presupposes in addition to an affective sense of identity with nature, pro-environmental attitudes and behavior, that is also indicated in a meta-analysis study (Mackay and Schmitt, 2019). This is a proposal that resonates with our model, as we have listed important characteristics that we seek to include in the levels of experience and activities to be developed. They are: the feeling of belonging; awe and reverence for the beauty, complexity, and diversity of nature; appreciation and respect; understanding and knowledge (a cognitive understanding of ecological processes, species diversity and conservation issues); and environmental awareness. Although the literature presents varied results regarding the magnitude and different levels of connection between people and nature, mediated by cultural, political, social, age, and other factors, the aspects that determine these variations remain unknown (Soga and Gaston, 2023). This knowledge gap is particularly evident in the Southern Hemisphere, where climatic and natural characteristics differ from those of the Northern Hemisphere, posing challenges in establishing parameters to prevent and reverse the disconnection from nature. Despite the increasing number of studies on the benefits of contact with nature on health (Frumkin et al., 2017), it is important to emphasize that contact with nature differs from connection with nature.

Contact with nature refers to the interaction between an individual and any element of the natural world or being in the presence of a natural environment (Garza-Terán et al., 2022). Human contact with nature can be classified as: (1) Indirect: involving contemplation of a photograph, a painting or even a real scene through a window, for example. Physical presence is not necessary; (2) Incidental: there is physical presence, but contact happens unintentionally, such as having a plant in a room; (3) Intentional: there is physical presence and the intention of contact with nature, such as choosing to hike or walk in a park (Keniger et al., 2013).

Therefore, our model is based on connection with nature, which has been shown to be more relevant to health and well-being than just contact with nature (Martin et al., 2020). It is more important to be with nature than just in nature.

A critical analysis of activities aimed at improving connection with nature suggests that activities based solely on knowledge, simple contact with green spaces such as parks, or a patio with vegetation, do not always lead to short-term improvements in nature connectivity (passive contact). There is evidence that active engagement with nature depends on the activity performed, as traditional outdoor adventure programs have not led to increased connection with nature, likely because the focus is on the challenge or adventure in nature, rather than a deeper relationship with nature itself (Richardson et al., 2020).

Indeed, studies have shown that activities that involve emotional attachment, meaningful experiences, and a compassionate relationship with nature are essential for increasing connection with nature (Lumber et al., 2017; Barragan-Jason et al., 2023). These types of activities may include things like gardening, nature walks, bird watching, and other activities that involve a deeper and more personal engagement with the natural world. Such activities can help individuals develop a greater sense of awe, wonder, and reverence for the natural world, leading to a more profound and meaningful connection with nature.

It is considered contact the act of engaging with nature through the senses for pleasure, for example: listening to bird songs, smelling wildflowers, watching the sunset. Beauty presupposes involvement with the aesthetic qualities of nature, such as appreciation of natural landscapes or artistic expression of nature. On the other hand, meaning implies using nature or its symbolism (e.g., language and metaphors) to represent an idea, thinking about the meaning of nature and its signs (Lumber et al., 2017; Pocock et al., 2023).

Emotion is represented by the emotional bond and love for nature, such as talking and reflecting on one’s feelings about it, which can contribute to its conservation. Lastly, Compassion seeks to extend the self to include nature, leading to a moral and ethical concern for nature, for example: making ethical choices of products, caring for plants and animal welfare (Lumber et al., 2017; Choque, 2021; Prato-Previde et al., 2022).

Additionally, it is important to emphasize that the pathway that operates in this dimension deserves to be explored not only during activities in nature, but permeating individuals’ lives even when they are not in a natural environment, like small doses, so that the experience lived in nature is not forgotten amidst the daily urban chaos for a significant portion of the population. However, promoting such integration is complex and challenging.

It is also worth noting that, beyond predictors of connection with nature, paths of connection, and recommended activities, two other aspects may be extremely relevant and have been little addressed or integrated in the literature. The first concerns natural characteristics that may affect the connection with nature differently when considering Kellert’s values (Kellert, 1993), such as fear that may accompany certain natural environments due to factual or imaginary risks. The second aspect is the diversity of nature-based health interventions that are labeled as forest bathing but, in fact, are not completely described, discussed or adapted for different contexts.

We have been reflecting a lot on forest bathing, a practice widely described in the literature that has also become popular in non-scientific circles. It is an activity originating from Japan that consists of experiencing the atmosphere of nature through the senses, associated or not with other integrative techniques such as meditation, Yoga, etc. (Li, 2019). Unlike Japan where it is mainly done in temperate forests or in Europe in coniferous forests, Brazil has a greater diversity of biomes, whether forest (Amazon rainforest or Atlantic Forest), cerrado, pampa, coastal strip, among others, that offer an experience that can vary due to the landscapes and faunistic and floristic biodiversity that compose them. Different aspects of the natural environment deserve future investigations and their relationship with the connection with nature. In countries with these characteristics, even though inspired by Japanese forest bathing, it might be more appropriate to use the term “nature bathing.”

On the other hand, the research protocols for nature-based health interventions are not well-defined in the literature, and there are few training programs available for conducting such interventions. These programs vary in terms of duration, theoretical framework, and program content. This highlights the need for creativity in designing intervention protocols, which should be aligned with the theoretical assumptions of interdisciplinary teams, their existing or developing skills and competencies, and be compatible with the reality in which they will be implemented.

## 2. The nature-based intervention model “A time with e-Natureza (e-Nature)”

This model arises from the perspective that health and nature are complex adaptive systems and is rooted in scientific literature, as well as the training and experience of researchers engaged in field activities within nature. These activities encompass various domains such as birdwatching, environmental education, nature photography and wildlife observation, appreciative gaze, and other techniques for contemplation of the natural world. Furthermore, despite the existence of theories that seek to explain the relationship between human beings and nature, this knowledge scattered in the literature needed to be articulated in a new perspective not only to benefit humans but also non-humans in the development of nature-based health interventions.

In this study, we have adopted the term “A time with e-Natureza (e-Nature)” for the model, as it derives from our “e-Natureza” interdisciplinary research group and directly reflects the core idea of spending time with nature. This term encapsulates the key concept we seek to explore. The proposed model addresses the various ways people engage with nature, encompassing both individual and autonomous activities, as well as collective and guided interventions that aim to enhance the connection with nature. Our investigation encompasses a wide range of research in this area to provide a comprehensive understanding of the subject.

The intervention model “A time with e-Natureza (e-Nature)” consists of a set of activities congruent with four pillars of support: (1) Aesthetic and emotional experience, (2) Multisensory integration experience, (3) Knowledge experience, and (4) Engagement experience, it represents a fundamental component of a nature-based intervention aimed at human health and well-being and nature conservation. It also draws significant inspiration from the Japanese practice of forest bathing, particularly in the activities outlined in pillars 1 and 2. However, two additional levels of experience have been integrated, recognizing their essential role in fostering not only human well-being but also biodiversity conservation. Considering the dynamic interaction among the elements involved in the intervention, including people, plants, animals, the natural environment, and the local culture, this model can be recognized as a Complex Adaptive System (CAS). Furthermore, this nature-based health intervention can involve multiple interactions and feedback loops among the elements, and these interactions can change over time in response to different stimuli and events. For instance, the natural environments where the intervention is carried out may be affected over time by factors such as climate change, resource availability and community demand. Additionally, any nature-based intervention may also have an impact on the natural environment, even if efforts are made to mitigate any potential harm.

**Figure 1:**
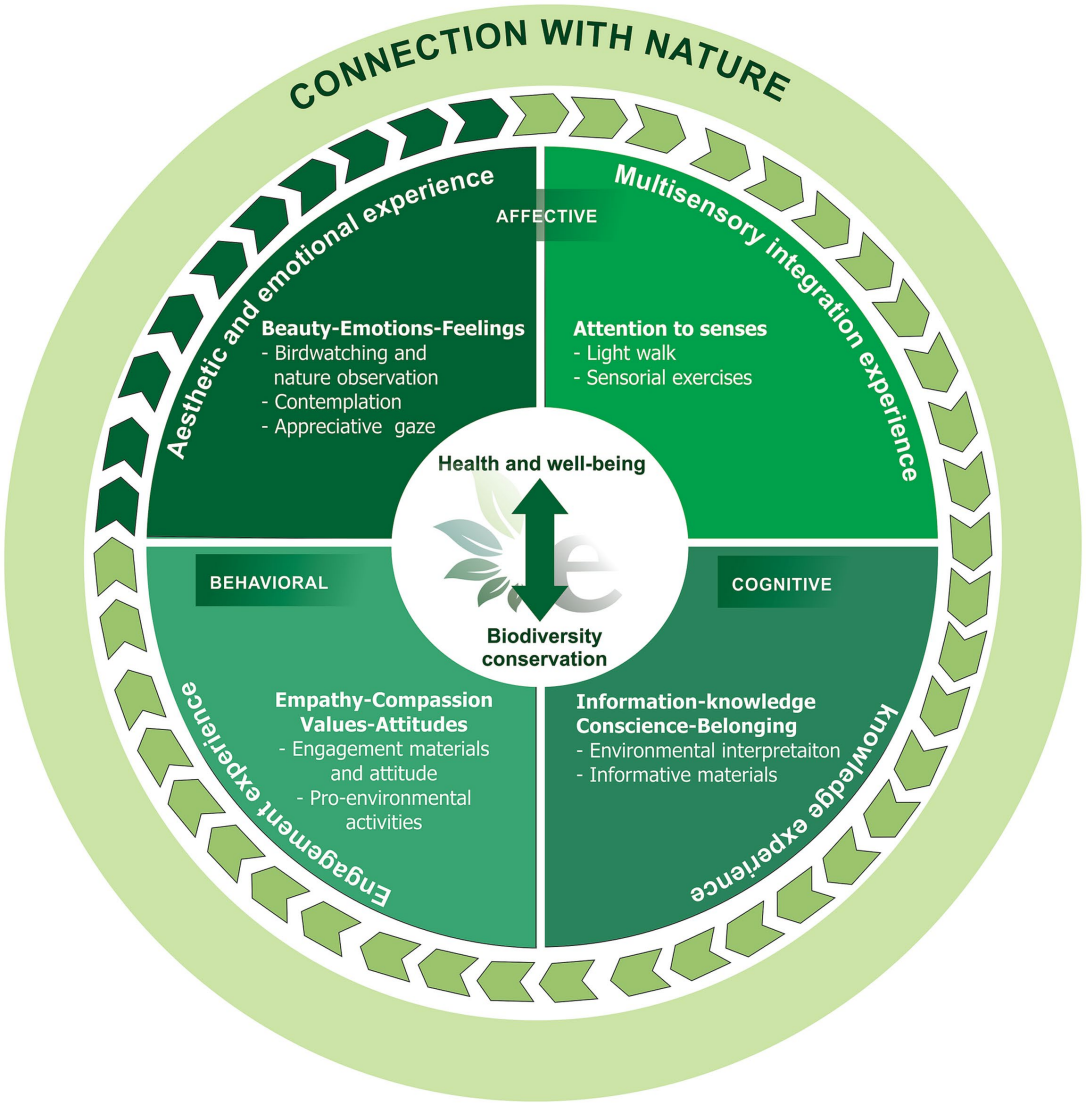
Diagram representing “A time with e-Natureza (e-Nature).”

Understanding this intervention (like any other that is proposed to be carried out in natural environments) as a CAS can help identify and anticipate potential impacts and improve its long-term effectiveness and sustainability. Figure 1 represents our model. As illustrated in the Figure, the model presents two expected outcomes for a nature-based intervention: improved health and well-being, and biodiversity conservation. The four types of experiences that make up the model are described in the subsequent circle. These experiences are: Aesthetic and emotional experience, Multisensory integration experience, Knowledge experience, and Engagement experience. The dark green circular arrow indicates the starting point of the model (Aesthetic and emotional experience) and the following arrows indicate the feedback loop that is created as the experiences interact with each other. The outer circle represents the connection with nature, which is developed through the integration of the four experiences. The Figure also depicts the three pillars of the connection with nature: the affective component, the cognitive component, and the behavioral component. The affective component is linked to the Aesthetic and emotional experience and Multisensory integration experience. The cognitive component is linked to the Knowledge experience. The behavioral component is linked to the Engagement experience.

### 2.1. The aesthetic and emotional experience – beauty/emotions/feelings

An aesthetic experience is a perceptual experience focused on the beauty of an object such as a work of art or an aspect of nature, but it is also possible to experience it not through perception, but through an emotional experience configured in sensations of pleasure with a positive hedonic tone (Peacocke, 2023). Aesthetic perception involves emotions and feelings. Emotions accompany and inform our experiences of art, literature, music, nature, or attractive visions, sounds, and lines of thought in general. Emotions such as being touched by something, beauty, fascination, captivation, reverence, transcendence, enchantment, and admiration (and also inspiration) are indicated in the literature as a state of appreciation that reveals dimensions of aesthetic appeal (Schindler et al., 2017). Visual stimuli of natural elements (not only landscapes) trigger different aesthetic reactions and levels of relaxation. Images of the sea, colorful birds, and pale birds have high valence, while images of flowers and the sky tend to be more relaxing (Dal Fabbro et al., 2021).

In the proposed model, this is of particular importance, as birdwatching during a nature-based health intervention has been one of the key tools to arouse interest, attention, but above all, to cause enchantment, generate empathy, and provide knowledge about nature, either literal or metaphorical. It is important to note that although didactically separated, the elements that make up the experiences are interchangeable.

Aesthetic experiences present different qualities: (a) they can reflect on the aesthetic value of an entity (i.e., resulting from engaging in aesthetic contemplation); (b) they can come to a decision about it (i.e., making an aesthetic judgment); or (c) they can involuntarily shift our attention to the aesthetic processing of a stimulus (i.e., experience of aesthetic distraction). However, some researchers have pointed out that there are experiences that operate in an “aesthetic mode,” which involves a disinterested interest, focusing attention on the stimulus, integrating context, memory, and sensory qualities, and neglecting self-referential concerns or perceptions of everyday life (Weigand and Jacobsen, 2021).

This aesthetic mode is related to Kaplan and Kaplan’s Attention Restoration Theory (Kaplan, 1995). This theory suggests that individuals require constant effort to avoid losing their focus on something more interesting. However, this daily effort to maintain concentration eventually leads to fatigue, which triggers a series of stress-related symptoms (Wang and Zhao, 2020). This theory is composed of four elements: fascination, being away, extent, and compatibility. Fascination is directly related to the aesthetic experience, as it presupposes the entry into esthetically pleasing stimuli that allow for the opportunity for reflection, promoting more efficient attention restoration (Kaplan, 1995). Researchers have shown that the more mentally fatigued a person is, the more likely he or she would choose a restorative walk in a natural environment over an urban. Study on preferences for four of the restorative components of natural environments proposed by Attention Restoration Theory found that these qualities are highly correlated with the aspects of an environment that independently make it a “favorite place” for individuals (Korpela et al., 2001). Indeed, a place that evokes pleasant sensations contributes to the creation of a sense of identity and connection, which, in turn, also results in restorative effects (Ratcliffe and Korpela, 2016). Exposure to the beauty of nature can increase the frequency and intensity of aesthetic experiences, which contributes to improved emotional abilities and greater life satisfaction by activating the brain’s reward system (dopaminergic activity – dopamine is a neurotransmitter responsible for relaying information to various parts of the body and when released, it triggers feelings of pleasure and increases motivation).

Aesthetic experiences are immediate, experiences of physically and visually (or multisensorially) pleasant environments that can help reduce stress, as they trigger positive emotions, maintain a non-vigilant state of attention, decrease negative thoughts, and allow for the return of physiological arousal to more moderate levels, as advocated by the Psychophysiological Recovery Theory to Stress (Ulrich, 1983).

Despite the fascination element being the one most directly related to the aesthetic experience, the other components of this theory (Kaplan and Kaplan, 1989) create the necessary conditions for this aesthetic experience to occur more easily. By moving away from everyday life (being away), being in contact with nature, feeling affinity with the natural environment (compatibility) and perhaps experiencing the feeling of belonging to something much greater (extent), it gives greater quality to the aesthetic experience, as well as increases the possibility of encountering various elements that provide the fascination, emotions and feelings associated.

#### 2.1.1. Proposed activities for the aesthetic and emotional experience

Engaging in various activities in nature can lead to aesthetic-emotional experiences. These experiences arise when individuals are captivated by the beauty, tranquility, and awe-inspiring elements of the natural environment.

##### 2.1.1.1. Birdwatching and nature observation

Birdwatching, also known as birding, is the activity of identifying and observing birds in their natural habitat. It can be carried out by anyone and can be done virtually anywhere in the world, from the densest forest to urban parks, and has been found to be effective in providing enchantment to observers. Thus, birdwatching can be combined with other outdoor activities such as hiking, camping, and nature photography, and in this study, it is considered an essential activity for intervention. Moreover, it is also recognized as a conservation tool, contributing to the monitoring of biodiversity and the protection of birds and their habitats.

In a study involving 26 European countries, the authors established a relationship between the diversity of bird species in a region and the self-evaluation of life satisfaction by residents of those regions (Stobbe et al., 2022). It was observed that a 10% increase in bird species diversity increases life satisfaction approximately 1.53 times more than a proportional increase in income (Methorst et al., 2021). If this occurs considering the regular presence of 540 species in Europe, differences in the experience can be expected in a natural environment such as Brazil, where 1,825 species occur, according to the Brazilian Committee of Ornithological Records, or in Colombia, the world record holder in bird species with 1,924 identified species, with its richness of sounds and colors.

In an online randomized experiment, 295 participants were exposed to different sounds for 6 min: low traffic noise, high traffic noise, low bird song, and high diversity bird song soundscapes. The results showed that the soundscapes of traffic noise were related to a significant increase in depression. Regarding bird song, depression decreased exclusively after exposure to the high diversity bird song soundscape (small effect size). In addition, both anxiety and paranoia decreased significantly in both bird song conditions (medium effect size). Therefore, the results suggest that listening to bird songs, regardless of diversity, improves anxiety, while traffic noise is related to greater depression (Ratcliffe et al., 2013).

##### 2.1.1.2. Contemplation and appreciative gaze

Nature contemplation is the practice of appreciating and observing the natural world, often in search of a deeper and more meaningful connection with the environment around us. This can involve observing landscapes, animals, plants, water and sky, or simply sitting quietly and paying attention to what is around us. Nature contemplation has been valued by many cultures throughout history as a way to promote inner peace, relieve stress, inspire creativity and reflection, and even improve mental and physical health.

Some scholars believe that contemplation of nature can be an important practice to help people reconnect with the environment and develop a sense of responsibility and care for the planet. Rachel Carson, a writer, and marine biologist who is famous for her book “Silent Spring,” which warned about the negative effects of pesticides on the environment, believed that contemplation of nature was essential to understanding the interconnectedness of all living beings and to developing an environmental ethic. Contemplative attention to the natural world is not only a spiritually significant act in itself, but can also generate ethical actions (Eggemeier, 2014).

The prevalence of colors such as green, brown, and blue in any forest environment, along with the contemplation of fractal patterns and shapes (such as branching trees, bushes, rivers), can convey specific visual stimuli to the central nervous system that humans have a habit of perceiving as “familiar” since ancient times (Antonelli et al., 2021). Additionally, the act of contemplation is often associated with aesthetic judgment (Di Dio et al., 2016).

Another possibility is engaging in nighttime activities, such as stargazing. A study demonstrated the concept of “Dark Nature,” which encompasses both the nocturnal environment and the nature-interaction activities available in it. The results indicated that participants with more years of stargazing experience and those who reported noticing wildlife during stargazing had higher levels of nature connectedness. The responses from participants suggest that stargazing can be considered a Dark Nature activity, as it involves interacting with the nocturnal environment, rather than simply taking place in the dark, and may offer similar benefits to those experienced by individuals participating in daytime activities in natural environments (Bell et al., 2014).

On the other hand, stargazing is an activity that can help mitigate the detrimental effects of light pollution, such as disruptions to biological rhythms and overall behavior of animals and plants, significant economic and energy losses (Mizon, 2002). The loss of dark skies influences not only our perception of the nighttime environment and physical health but also our overall well-being (Bjelajac et al., 2021).

### 2.2. The multisensory integration experience – attention to senses

Multisensory integration is the brain’s ability to combine sensory information from different sensory channels, such as vision, hearing, touch, smell, and taste, to form a more complete and accurate perception of the environment. In other words, it is the capacity to integrate sensory information from multiple sources to form a more complete and more accurate picture of the world. Multisensory integration not only exerts bottom-up control, but also top-down control over attention. This ability is essential for many cognitive tasks, such as object recognition, spatial orientation, decision-making, and social interaction. In the intervention “A time with e-Natureza (e-Nature), multisensory integration arises both from endogenous attention, also called voluntary or goal-directed attention, when the facilitator conducting the activity draws attention to some element of the natural world, such as a flower or bird observed nearby, involving a more purposeful and effort-intensive process of orientation (Macaluso and Maravita, 2010). But it also makes use of exogenous attention, also called involuntary or stimulus-driven attention, which can be reflexively triggered by a salient sensory event in the external world (Hopfinger and West, 2006), such as the participant having their attention drawn to a bird landing on a tree, even if the facilitator did not draw attention to it.

Integrated multisensory events can efficiently capture attention even in complex circumstances, due to their greater relevance compared to unimodal events, and therefore can enhance the experience. Moreover, within a multisensory experience, endogenous attention can spread from one modality to another in an exogenous manner.

#### 2.2.1. Proposed activities for the multisensory integration experience

Proposed activities for the multisensory integration experience aim to engage multiple senses and enhance our connection with nature. These activities involve immersing ourselves in the sights, sounds, smells, textures, and even tastes of the natural environment.

##### 2.2.1.1. Light walk

Engaging in physical activity is often associated with physical and mental health, and exercise performed in natural environments has been considered more effective and enjoyable. Researchers have demonstrated that a gentle 10-min walk covering approximately 400 meters in a natural environment is sufficient to influence cognitive test outcomes (Bailey and Kang, 2022). In this context, connection with nature leads individuals to experience more mindfulness during the outdoor intervention, as well as reducing rumination as evidenced by a lower frontal beta wave during the experience. This reinforces the Attention Restoration Theory and the effectiveness of short outdoor interventions incorporating physical activity as a method of restoring mental attention (Bratman et al., 2015).

Regular physical activity contributes to the primary and secondary prevention of various chronic diseases and is associated with a reduced risk of premature death (Warburton et al., 2006). The proposed model includes a short and light walk to emphasize the relationship with the natural environment, but it is still a fact that exercise is present and can be intensified if the participant perceives benefits to their health, making it a regular practice (Lahart et al., 2019). A study with almost 30,000 participants demonstrated that promoting any type of walking can be a way to help adults avoid inactivity and encourage an active lifestyle for the prevention and treatment of cardiovascular diseases (Omura et al., 2019).

##### 2.2.1.2. Sensorial exercises and appreciative gaze

The appreciative gaze is understood as a mindfulness practice in which, through an integrated multisensory approach, the individual consciously connects with elements of their own reality, resulting in a sense of well-being (Isanta, 2018). The practice of the appreciative gaze involves the integration of all senses in the search and recognition of elements available in the present moment that can be a source of appreciation, satisfaction or enchantment when rescued from their pseudo-invisibility, a state in which they would remain if not consciously perceived and valued (Fingerhut and Prinz, 2018).

It is a trainable and reproducible mental attitude (Kolb and Whishaw, 1998). With regular repetition of small appreciative gaze practices distributed throughout the day, elements in the environment can be found and processed as sources of enchantment. The awakening of enchantment, in turn, has the potential to trigger the endogenous release of substances responsible for the feeling of well-being (Creswell, 2017).

The perception of reality results from subjective and intransferable filters, involving genetic, sociocultural, environmental, and emotional components, among others. However, the appreciative gaze, with its sensorial exercises, generates a personal satisfaction response that transcends differences and contributes to the feeling of well-being (Lyubomirsky et al., 2005; Seligman, 2011), potentially representing an accessible tool for stress management (Menardo et al., 2022).

### 2.3. Knowledge experience

A knowledge-based experience in nature is one in which a person gains access to a range of information about the natural world. During this experience, participants can observe, explore, and learn about different aspects of nature, including biodiversity, ecosystems, natural processes, and the challenges faced in environmental conservation. The aim of a knowledge-based experience in nature is to deepen understanding and connection with the natural world, fostering a sense of appreciation, awe, and responsibility towards the conservation and preservation of nature.

#### 2.3.1. Information/knowledge

When discussing knowledge, it extends beyond merely providing information about the natural environment where nature-based interventions occur. Knowledge also relates to Aesthetic experience. It is important to keep in mind the “stop for knowledge” hypothesis, which refers to learning something new. Aesthetic experiences cannot be reduced to a mere decorative aspect of life; they must be considered a fundamental part of our knowledge acquisition process. This requires working memory resources, and research evidence and neuroimaging show that beauty is associated with motor inhibition as well as increased attentional focus on sensory stimulation. This concentration of processing resources on the object of aesthetic appreciation is crucial for learning. The presence of aesthetic experiences keeps observers focused on the constantly changing present moment and intensifies the sensations and emotions provoked by the beauty of what is being observed. This allows observers to direct their attention to the perceptual activity, i.e., the experience of knowledge, which is also related to the multisensory integrated experience, resulting in sensory amplification. There is evidence suggesting a connection between aesthetics, pleasure, and perceptual learning, which aims to update the predictive mental environment (Sarasso et al., 2020).

The process of adaptation is crucial for optimal learning, enabling better interaction with the external world and modulation of our behavior. In simple terms, the beauty of nature arouses in us a curiosity for novelty, as even though it is made up of recognizable patterns, it never repeats itself. Depending on the time of day, the lighting, and other factors, nature constantly presents itself to our perception as renewed (Sarasso et al., 2020).

Activities related to education and environmental interpretation as a way of enhancing aesthetic experiences can provide knowledge that leads to behaviors more connected with nature. This knowledge is fundamental for Engagement experience to be effective. It is important to highlight that lifelong education is built upon four fundamental pillars: learning to know, learning to do, learning to live together, and learning to be. These pillars enable us to embrace the wisdom shared by African conservationist Baba Dioum, who emphasized that ultimately, we will preserve only what we love, love only what we understand, and understand only what we are taught.

#### 2.3.2. Conscience and belonging

The sense of belonging brought to consciousness can also be achieved by promoting connection with nature and consequent pro-environmental behaviors (Stern, 2002). According to a meta-analysis, there is a strong association between connection with nature and pro-environmental behaviors, such that the more an individual feels connected and part of nature, the more they promote conservation actions. A brief artificial exposure to elements of nature, using images and videos, may not be strong enough to enable such a sense of unity and connection (Mackay and Schmitt, 2019).

The sense of belonging can be extrapolated to other forms of connection. Connection with humanity, understood as the link with people outside of family and friend’s groups, and the transformation of this bond into a sense of familiarity with other inhabitants of the world, generates the concept of global belonging, altruistic and conservationist attitudes (Buchan et al., 2011; Der-Karabetian et al., 2014). However, making this sense of belonging conscious seems to be a current challenge. One of the factors is the constant reduction in the time children spend outdoors and exposed to nature, to the detriment of more screen time (Kellert et al., 2017), which can lead to the phenomenon called “Nature Deficit Disorder” by Louv (2008).

As a strategy to change this scenario of children’s relationship with nature, Social Emotional Learning (SEL) of children has been valued. SEL is defined by the Collaborative for Academic, Social, and Emotional Learning (CASEL) as the process that enables the acquisition and application of competencies directed towards social awareness and interpersonal relationships (Collaborative for Academic, Social, and Emotional Learning, 2022). Recently, a relationship has been demonstrated between nature connection and such competencies, such that the development of nature-based SEL interventions can facilitate and strengthen children’s psychological connection with nature (Lanza et al., 2023).

The role of belongingness and awareness in this model’s Knowledge Experience is therefore to drive connection and provide experiences with content and natural environments, breaking a cycle of lack of knowledge – reduced experiences in natural environments – negative emotions and behaviors directed towards nature (Soga and Gaston, 2016).

Hence, the creation of spaces and activities, such as the one presented here, becomes crucial in fostering a deeper connection between individuals and nature. These endeavors aim not only to offer insights into the health and well-being benefits supported by scientific literature but also to provide information and knowledge about the natural world. By doing so, they contribute to strengthening environmental awareness and nurturing a sense of belonging. It is our belief that these efforts will foster shifts in attitudes and transformative changes that hold significance in the lives of every individual involved, ultimately enhancing the well-being of all, and promoting planetary health. Studies show that the sense of kinship, egalitarianism, incorporation, and belonging associated with a strong connection with nature promotes high levels of well-being (Mayer and Frantz, 2004). In addition, the innate emotional affinity that humans have for nature and other forms of life, such as pets, often translates into positive emotions when they are in the natural environment (Nisbet et al., 2011; Zelenski and Nisbet, 2014). Other authors (Mayer et al., 2009) argue that experiential feelings of belonging to nature facilitate people’s ability to have purpose and meaning in their lives. In other words, the connection with nature can be a key factor that favors positive psychological functioning, as pointed out in an excellent current meta-analysis that investigated the relationship between nature connection and eudaimonic well-being (Pritchard et al., 2020).

#### 2.3.3. Proposed activities for knowledge experience

Engaging in knowledge experiences in nature involves actively seeking and acquiring knowledge about the natural world. By immersing ourselves in these knowledge-based activities, we can deepen our understanding of the natural world, appreciate its complexity, and develop a stronger sense of connection and appreciation for the environment. These experiences not only expand our knowledge but also foster a deeper sense of wonder and respect for the intricate web of life that surrounds us.

##### 2.3.3.1. Environmental interpretation

Environmental interpretation is the practice of communicating information about the natural and cultural environment of a specific area to the general public, with the aim of increasing awareness, understanding, and appreciation of the environment and its importance to society. It can be considered an important tool for environmental education (Ham, 1992). Environmental interpretation usually involves presenting information about the ecology, history, geology, culture, and other aspects of the natural and cultural environment, using a variety of methods including interpretive trails, informative signs, etc. Based on Tilden’s principles, environmental interpretation should be enjoyable, meaningful, organized, thought-provoking, distinctive, and thematic (Tilden, 2009).

The goal is to engage and inspire the public to become more involved and responsible for the conservation of the environment. The proposed intervention adheres to the methodological recommendation of Environmental Interpretation, which advocates for a prior preparation in the field regarding the physical and discursive environment to be explored, with the goal of making it an event capable of capturing the individual’s attention and reflection during the visit. This is necessary because the degree of influence of Environmental Interpretation depends on how the messages are formulated and presented to the audience. Increasing the degree of attention on the environment, favors of the state of presence, which fosters an appreciative gaze and pro-environmental behavior by strengthening the sense of belonging (Ham, 2007).

##### 2.3.3.2. Informative materials

In addition to the knowledge shared during the execution of the intervention, structured materials can offer qualified information that helps in the expansion of this knowledge by the participants, as well as can help in the engagement after carrying out the nature-based intervention, contributing to an active experience.

### 2.4. Engagement experience

An experience for engaging with nature is one in which a person actively participates in physical, sensory, and intellectual activities that involve direct or even indirect interaction with the natural environment. By actively engaging with nature, individuals can develop a greater appreciation for biodiversity, understand ecosystems, and become active advocates for environmental conservation. To achieve this, it is essential to reflect on the intrinsic value of nature, beyond the ecosystem services it provides.

#### 2.4.1. Empathy/compassion/values/attitudes

Engagement experience is based on the relationship between beings permeated by empathy, compassion, and values. Empathy is a concept that has been widely explored but without absolute consensus. In this model, empathy is conceptualized as comprising three pillars: (1) affective, wherein individuals can experience and understand the emotions and perceptions of others; (2) cognitive, involving the comprehension of another person’s situation; and (3) behavioral, encompassing the expression of one’s understanding and perceptions to assist and support others (Eklund and Meranius, 2021).

In this perspective, empathy extends beyond mere perspective-taking and involves a comprehensive understanding of another person’s situation. It also emphasizes the importance of communicating this understanding to the individual, thus creating awareness, and subsequently, motivating helpful actions. In other words, empathy is a step that precedes compassion. Not by coincidence, the same three pillars compose the Connection with Nature. It is possible to intersect them to apply the concept of empathy not only to human beings, but to all living beings and nature itself.

In the US, there is already research on criteria for green areas to evoke empathy in their visitors. One of the American examples that stands out is the Hillside Environmental Education Park (HEEP), located on University of Connecticut (165 acres of conservation area located on the North Campus of UConn). HEEP is publicly owned and focuses on nature; is one of the few designs in the United States that integrates human interactivity, processes, and interpretive learning —a practical hotbed for affective empathy. HEEP results found that green infrastructure projects can facilitate empathy with nature in their users. If projects allow (or even facilitate) the experience of empathy with nature, they can result in promoting a slow change in attitude that can lead to a change in behavior in the way people interact with nature. People would begin to see nature as a meaningful and validated other that they can understand and feel compassion for (Matthias, 2020).

Another example of the application of an affective method of environmental education, applied to third grade students from an education unit, in the city of El Alto, La Paz-Bolivia, is directly related to the four levels of the proposed model, not directly citing the aesthetic experience that seems to be implied, as it is an experience with ornamental plants. This method, developed under the focus of environmental psychology and environmental education, aims to promote affective bonds with nature. The affective method consists of three phases: the cognitive, affective, and active phases. The first is to spread the theory about the importance of care for nature, the ecosystem and plant care guideline ornamental. The affective and active phase begins with the adoption of ornamental plants, and with the execution of activities such as the elaboration of a newspaper, shower of plants, letter to the plant, among others; with the aim of developing a relationship affective relationship between students and their plant as a tool for learning and developing pro-environmental behaviors (Choque, 2021).

Indeed, research suggests that emotions are crucial for ecological knowledge to translate into pro-environmental behavior. Empathy seems to be the initial stimulus to generate such emotions, in conjunction with the provision of information and knowledge (Myers et al., 2009).

This cycle of empathy-emotion-knowledge–behavior is used in our model, even though empathy is didactically located in the Engagement experience, it permeates the other Experiences as a potential trigger. The directed empathy behavior towards situations of suffering, both for humans and animals, has shown to influence pro-environmental arguments, so that incentivized empathy can evoke arguments and, consequently, conservation-related behaviors (Berenguer, 2010).

Therefore, empathy can be a tool for promoting and expanding the connection with nature, culminating in environmental preservation behaviors. Such conservation-oriented attitudes can be seen as the result of compassionate behavior towards other non-human living beings, so that for some authors, compassion is the next step after empathy, as it presupposes, by definition, an action aimed at alleviating the suffering of others (Goetz et al., 2010). Therefore, compassion has been considered a path to conservation, based on the Moralistic value of Biophilia (Lumber et al., 2017). In the context of our model, we recognize that empathy is a prerequisite for compassion. We believe that for a compassionate attitude to be fully realized, it must be preceded by the practice of empathy.

Values are the set of characteristics of a particular person (or organization) that determine how they behave and interact with other individuals and the environment. In the contemporary world, nature has been conceived as a set of objects that are recognized or valued based on people’s perceptions. Values are provided by humans, and their most common expressions are, for example, the attribution of economic value to some natural resources or the attribution of property rights over green spaces (anthropocentric view). On the other hand, since the 1960s, another perspective recognizes that Nature has certain values that are inherent to it (non-anthropocentric view), regardless of its usefulness or potential benefit to humans (Gudynas, 2010).

**Figure 2:**
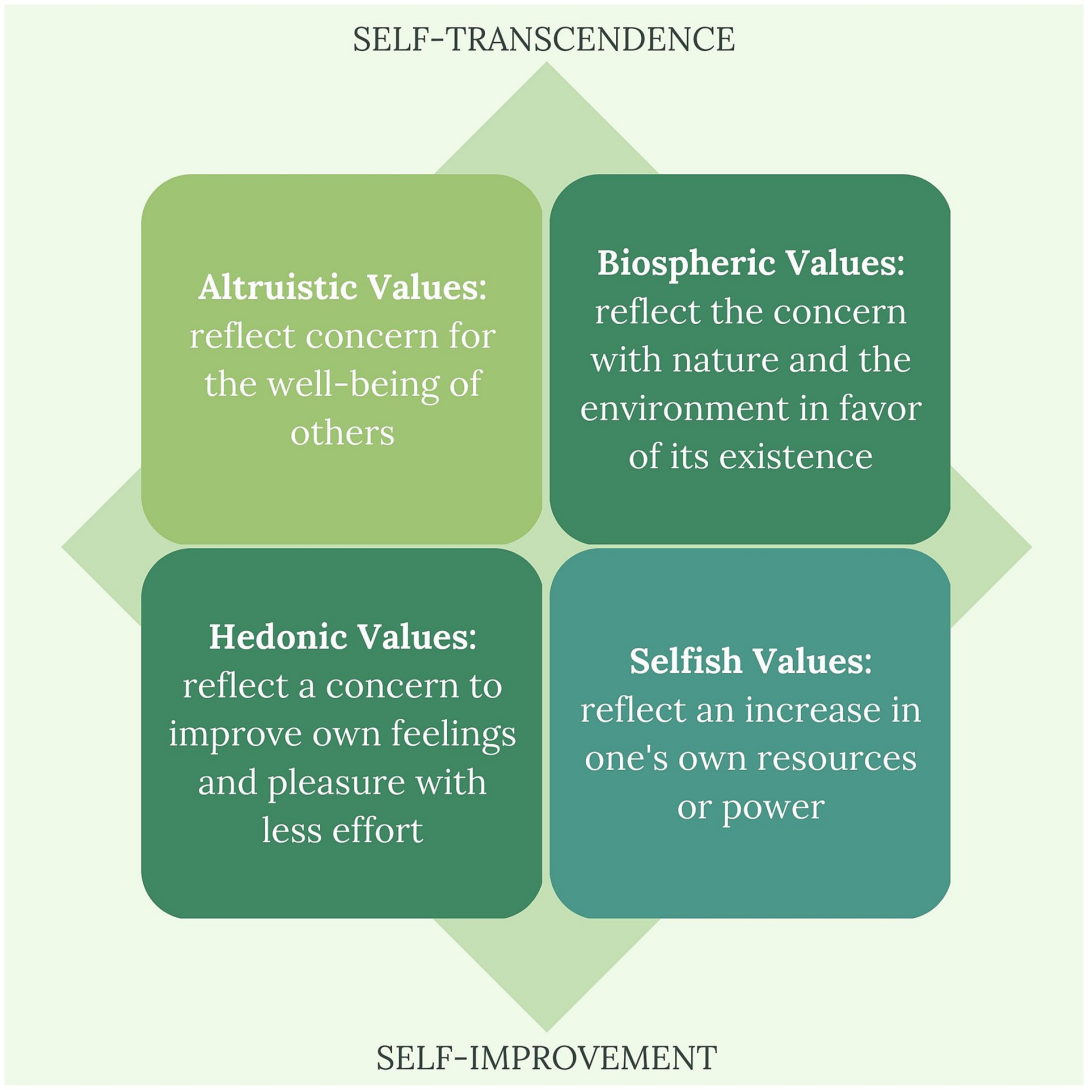
The values of self-transcendence and self-improvement that influence the relationship between humans and nature.

The values can also be classified as self-transcendence or self-improvement (Steg et al., 2014), as presented in Figure 2.

The anthropocentric view can be instrumental or relational. In the instrumental view, the focus of value lies in the contributions that nature has to offer for people (creation of habitats, food, energy, ecosystem services, materials). In the relational view, in which nature-based health interventions fit, the focus of value lies on the good quality of life (physical and experiential interactions in nature, symbolic meaning, inspiration, physical, mental, and emotional health, cultural identity, social cohesion). In the intrinsic (non-anthropocentric) view, the focus of value is entirely directed towards nature, remembering that humans are also part of it (but without dichotomy), and there is a possibility of well-being that includes all beings, with respect to the Earth, evolutionary and ecological processes (DeVille et al., 2021), and the conservation of biodiversity, on which all depend on the planet.

Our aim is to highlight this concern in the outermost circle of the model, underscoring the inseparable connection between nature, health, well-being, and nature conservation. We firmly believe that these elements should be integrated into any nature-based health intervention.

#### 2.4.2. Proposed activities for engagement experience

These activities encompass a diverse range of options, including informative materials, engagement actions utilizing digital resources, and artistic practices. These activities are designed to actively engage individuals with the natural world, fostering a deeper connection and understanding of its intricacies. Informative materials, such as educational resources and guides, provide valuable knowledge about the environment, ecosystems, and wildlife, empowering individuals to make informed decisions and take responsible actions. Additionally, engagement actions utilizing digital resources, such as interactive apps, virtual reality experiences, or citizen science platforms, offer innovative ways to explore and contribute to environmental research and conservation efforts.

##### 2.4.2.1. Engagement materials and attitude and pro-environmental activities

In the proposed model, a series of activities with nature or activities that direct individuals’ attention to nature are adopted as possibilities for engagement actions. These activities are compiled in the Inspiration Guide on Nature-Related Activities^1^, organized by a multi-professional team, including mental health (Leão et al., 2023a), and in the Eco-Challenges Guide (see footnote 1) created by some of the authors of this article (Leão et al., 2023b), to maintain attention on pro-environmental actions. Both guides are available in electronic format and aim to sensitize and/or strengthen the connection with nature for moments when participants in nature-based interventions have returned to their daily lives. Among them, there are indications to explore the relationship between nature and art, nature sounds and images, and activities related to the proposed themes.

Digital technologies can also contribute to arousing people’s interest in nature and, consequently, stimulating this contact, which is why they are included in the inspiration guide. Some of the resources presented in this material that deserve special mention in this area include the iNaturalist platform, which can be accessed through the website https://www.inaturalist.org or through the iNaturalist application, for Android or iOS (California Academy of Science, National Geographic Society, 2023) and the Wiki Aves platform, which can be accessed through the website www.wikiaves.com.br/ (WikiAves, 2023).

The iNaturalist platform combines technology with nature, allowing users to record, learn, and share their nature discoveries with the community during a walk in the park, for example. On the other hand, the Wiki Aves platform provides a literal Wikipedia about various bird species, making bird watching a more natural and approachable activity for people’s daily lives.

In general, the digital technologies that dominate our daily lives, especially those that keep us connected to screens for long periods of time, are associated with factors that distance us from nature. However, currently, there are digital resources whose purpose is to stimulate and promote our contact with nature and contribute to scientific research through Citizen Science.

In addition, to strengthen the educational content (considered a key element for engagement), these materials direct users to other resources for sensitization and qualified information, such as the social media account @umtempocomenatureza, which presents posts about nature and health in Portuguese and English. On this platform, the sharing of experiences in nature, photos, and experiences with the hashtag #umtempocomenatureza are encouraged, considering that photography has become a great resource for sensitization and also for contemplation, and nature images have the potential to promote positive emotions when in non-natural environments (Dal Fabbro et al., 2021). A website that gathers information, commented scientific articles, book tips, music, etc. (Um tempo com e-Natureza, 2022) is also recommended as educational material to favor engagement actions.

While some people seek to learn about the nature that surrounds them, others seek to understand and even measure the benefits that connection with nature brings to health through digital resources. In the United States, the Nature Quant platform (NatureQuant, 2023) measures users’ exposure to nature during exercise, and based on their diet, sleep quality, physical activity, and exposure to nature, it can create a personalized nature prescription for the user. This increases the individual’s autonomy in seeking new ways to increase exposure to natural environments and thus promote connection with nature.

Lastly, artistic practices, such as nature-inspired photography, painting, or poetry, allow individuals to express their personal experiences and emotions, creating a bridge between art and nature. By embracing these proposed activities, individuals can actively immerse themselves in the beauty and importance of the natural world, can take care of your health and well-being and promote a deeper connection and inspire a sense of responsibility for the preservation of nature.

The arts and creative expressions have been used to enhance educational programs that seek to promote connection with nature (Bruni et al., 2015), just as our model encourages participants to seek to photograph, draw, write, among other possibilities to anchor their experiences in nature. Social prescriptions can be encouraged after the intervention has taken place and can include arts-based activities that operationalize pathways that continue to promote participants’ connection to the natural world (Richardson et al., 2020).

## 3. Discussion

In this study, we approach our proposal for a health intervention model based on nature, anchored in the concept of CAS, consisting of four types of experience that start from the interdependence of human health and well-being with the conservation of biodiversity to achieve a greater degree of connection with nature.

The activities that may compose a nature-based intervention for health and well-being can explore one or more paths that have been presented for each of the experiences that comprise the model. Even with better-established protocols, considering again that it is executed in CAS complex adaptive systems, which are open and self-organized, it is likely that one level of experience or another will be more predominant. This dependency relies on the characteristics of each individual who is participating or conducting the intervention, as well as the characteristics of the natural environment where it develops. Additionally, dynamic, emergent, and unpredictable factors further contribute to this complexity. Even with better-established protocols, considering again that it is executed in CAS complex adaptive systems, which are open and self-organized, it is likely that one level of experience or another will be more predominant, as it depends on the characteristics of each individual who is participating or conducting the intervention, as well as the characteristics of the natural environment where it develops and dynamic, emergent, and unpredictable factors. However, these four dimensions of experience should be integrated because the more the intervention is directed towards addressing them, the results may be more effective and long-lasting, which we are investigating.

The potential of nature to impact various aspects of human life is widely recognized. Nature provides us with essential resources such as food and raw materials, but it also profoundly influences our physical and psychological well-being through human interaction with the natural environment. In this model, human health and well-being are regarded as inseparable components of nature conservation.

The Intergovernmental Science-Policy Platform on Biodiversity and Ecosystem Services (IPBES) is a consortium of various countries (including Brazil) and organizations, headquartered in Europe and supported by the United Nations Environment Programme (UNEP). Its objective is to strengthen the interface between scientific policies and ecosystems for the conservation of global biodiversity, considering human well-being and sustainable development. The IPBES coined the term “Nature’s Contributions to People” (NCP) to exclusively describe such influences and benefits of nature to us (Díaz et al., 2018).

In the proposed model, environmental interpretation focused on key elements present in various natural environments was considered, as biodiversity plays a significant role in individuals’ experiences. For example, bird watching was not only based on the authors’ experiences, mostly bird watchers (including one ornithologist), but also its potential to evoke empathy and admiration (for beauty), provide the possibility to explore naturalist knowledge about birds, which goes beyond the aesthetic experience.

In addition, researchers (Methorst et al., 2021) have used indicators capable of quantifying natural aspects with ecological measures of species diversity as influential factors for human well-being, still, it seems that the abundance of bird species is as beneficial to the perception of satisfaction as financial abundance. This is another argument for reflecting on values that really matter. Interestingly, the same research’ results did not show a correlation between mammal diversity and satisfaction, despite the well-known positive emotional response aroused by large mammals that always seem so charismatic. The explanation for this seems to be in the low encounter rate of city dwellers with these mammals, which tend to avoid urban environments, while small mammals are nocturnal and difficult to see in general. Birds, on the other hand, are perceived by humans even if they do not see them, since their songs are easily picked up by our hearing, in addition to being diurnal and often very active, even in urban areas. The other surprise revealed by this research is the absence of correlation between tree diversity and the perception of satisfaction and well-being. This result seems to suggest that it is the overall structure of the vegetation, and not the trees themselves, that can indicate natural diversity and result in the well-known positive relationship with well-being.

A recent study that explored the inner and outer values on the conservation attitudes of farmers from an Amazonia deforestation frontier (Mikołajczak et al., 2023) evidenced that inner motivations are capable of nurture pro-environment behaviors, based on self-transcendence and self-improvement values. Although extern factors, like poverty, material and health insecurity and less years of formal education may support economic development over nature conservation. In this way, we reinforce the need for a joint development among different areas like economic, social, health and environmental to achieve a common goal.

Lastly, but not less importantly, it is worth noting that the proposed intervention model is related to the main theories that have underpinned the human-nature relationship. Some theories seek to explain how nature-based interventions impact human physiology, pathophysiology, and influence various systems, such as the immune, endocrine, cardiovascular, and nervous systems, as well as explain these effects, such as the Theory of Physiological Recovery from Stress (Ulrich, 1983) and the Theory of Attention Restoration (Kaplan, 1995). To classify an intervention as healthcare, it requires articulation with other theoretical assumptions that extend beyond explaining its potential mechanisms. In this regard, Nursing is an inspirational model since it is a science centered around human care and encompasses the prescription of interventions for health promotion. By incorporating the principles and practices of Nursing, our aim is to ensure a comprehensive and holistic approach to health within the proposed model.

Florence Nightingale was the precursor of modern nursing and developed the Environmental Theory, dated from 1859, which is still considered current and relevant. According to her, any external environment to human beings (including nature) is capable of interfering in the health-disease process, that is, the promotion and/or recovery of health are directly influenced by the environment in which an individual is inserted (Medeiros et al., 2015).

Therefore, it is a fundamental part of care to consider that human beings are part of nature and, therefore, when the environment is unhealthy, human beings are also unhealthy. In practice, Florence left a legacy about the role of a caregiver as the one responsible for maintaining the external environment as balanced as possible so that the internal environment (human being) can regenerate. Thus, keeping well-ventilated, clean, and well-lit areas, and offering a greater sense of well-being through connection with nature can contribute decisively to achieving greater health (Medeiros et al., 2015; Riegel et al., 2021).

Another theorist is Margaret Jean Watson, who in 1979 completed her first theoretical reflection and still today continues to refine and teach her way of understanding the role of nursing in care, called the Theory of Human Caring (Tonin et al., 2017). Its application is based on the premise of offering transpersonal care that is capable of surpassing the punctual moment of encounter between caregiver and cared for, with a focus on a common goal: the promotion of regeneration through the Clinical Caritas Process, which contains 10 fundamental elements that must be considered at the time of interaction and are based on the permanent belief that any being in need of care is a member of the universe and deserving of respect (Savieto and Leão, 2016; Watson Caring Science Institute, 2023), which includes the planet itself. Among them are the formation of a humanistic-altruistic value system, the cultivation of sensitivity to oneself and others, the systematic use of the scientific method of problem-solving to make decisions, the provision of a mental, physical, sociocultural, and spiritual supportive, protective, and/or corrective environment, and the acceptance of existential and phenomenological forces, considering that the care environment is the one that offers potential development while allowing the person to choose the best action for themselves at a given moment.

From the perspective of complex adaptive systems, Martha Elisabeth Rogers’ Humanistic and Humanitarian Theory can be adopted as the background for the prescription of “A Time with e-Natureza (e-Nature).” For her, nursing is understood as the science and art of promoting interaction between human beings and nature, aiming at maintaining their integrity and directing standardization. This theory is based on the principles of integrality, resonance, and helicity, as well as the concepts of energy fields, such as open systems (in constant exchange). Therefore, it is not possible to separate the understanding of the human being from their environment. The focus of this theory is the relationship between the human being and their environment, and the professional will have their action guided by this exchange, seeking an adequate therapy to provide conditions for pattern reorganization, intervening both in the environment and in the human being. Rogers’ theory reinforces the assumption that the human being, contrary to what is often observed, is not an isolated entity. Building upon this concept, she establishes her interventions, recognizing that the human being is a dynamic process of mutual interaction between the individual and the environment, considering various variables involved. It also considers that the identification of individuals and the reflection of their totality are life patterns that allow self-regulation, rhythm, and dynamism, providing unity to the diversity that reflects a creative and dynamic universe (Rogers, 1970). The very idea of biophilia originates from an understanding of evolution, whereby for over 99% of our species’ history, biological development occurred in adaptive response to natural forces that were non-artificial or human-created, and thus within complex adaptive systems.

To assess the practical applicability of this model in healthcare, for example, we can consider the NANDA International (NANDA-I) classification systems for nursing diagnoses, the Nursing Interventions Classification (NIC), and the Nursing Outcomes Classification (NOC) (Fennelly et al., 2021).

For this nursing taxonomy, the proposed model can be useful for nursing diagnosis (Herdman et al., 2021) such as stress overload, characterized by excessive amounts and types of demands that require action, or anxiety, characterized by a vague and uncomfortable feeling of discomfort or fear, accompanied by an autonomic response whose source is often non-specific or unknown to the individual. It can also be useful for other nursing diagnosis: health promotion, characterized by clinical judgment regarding motivation and the desire to increase well-being and achieve human potential for health. Health promotion responses can exist in individuals, families, groups, or communities. The intervention can also be applied to the diagnosis of Improved Self-Care Capacity, which involves engaging in activities for oneself to achieve health goals and can be strengthened (Herdman et al., 2021).Regarding intervention, the model can be framed within the NIC in the Environmental Management intervention, which assumes the optimal use of the environment for therapeutic benefit, sensory appeal, and psychological well-being, or in Health Education, which involves developing and providing individual or group instruction and learning experiences to facilitate voluntary adaptation of health-related behavior in individuals, families, groups, or communities, such as the materials that make up the proposed engagement experience in the model (Butcher et al., 2020).

However, it is necessary to recognize the effectiveness of the proposed intervention. The systematic use of outcomes to evaluate healthcare began when Florence Nightingale recorded and analyzed healthcare conditions and patient outcomes during Crimean War in the 19th century. Efforts to evaluate medical practice began in the early 20th century when Codman, a surgeon from Boston, proposed using outcome-based measures as indicators of the quality of medical care (Riegel et al., 2021).

Therefore, for outcome evaluation, the NOC taxonomy provides for the use of scales, such as the measurement of personal well-being, which are related to diagnoses of health promotion and well-being. The lexical and taxonomic development that provides standardized terms in a constructed classification stimulates the formulation of an inductive theory and the empirical testing of deductive theories. And through the accumulation of research results supporting the effects of interventions on patient outcomes for specific diagnoses, among other patient characteristics, evidence-based practice protocols can be developed, therefore these taxonomies were considered in this study (Moorhead et al., 2020).

Indeed, the establishment of diagnoses and the evaluation of outcomes encompass a wide range of validated scales for health outcomes, extending beyond the NANDA-I classification system. However, it serves as a backdrop to indicate that this rationale should be considered in the implementation not only of the model presented in this article but also of others proposing diverse nature-based interventions.

Looking to the future, interest in forms of indirect contact with nature in three-dimensional, immersive, and non-immersive virtual environments is growing. A project carried out with sounds of nature for recovery from intentional acute stress using a virtual tool called TSST, in which autonomic and endocrine stress responses were explored along with subjective assessments. The participants were submitted to such an experience and later to virtual reality (VR) with digital natural environments. The volunteers were divided into three groups, one with virtual forest and sound, one with virtual forest without sound and a control without virtual forest or sounds. Natural sounds of birds and water were used due to their beneficial properties mentioned in the literature. The study demonstrated that sounds of nature facilitate recovery from stress when used in green virtual environments indicated by the recovery of cardiac regulation, contributing to the increase of parasympathetic activity (Annerstedt et al., 2013).

Although our model was primarily designed to support interventions involving direct contact with nature, it is possible to develop interventions based on the model that utilize indirect contact through immersive reality. However, it is important to be aware that such interventions may have limitations compared to direct contact, as there are inherent differences and unique benefits associated with direct contact with nature. It is worth noting, however, that virtual experiences should not replace real experiences, but can be useful in contexts where they are not possible, as for hospitalized patients. Activities proposed in the model can be recorded, such as a birding-watching activity in a natural environment, environmental education content can be inserted, and technologies can be developed that will expand the multisensory and interactivity experience, beyond hearing and vision that a 3D experience normally allows in which some device of odor in the environment can be associated. It is, however, an intervention that will have to be tested in relation, mainly, to the Engagement experience that the model proposes, which already configures the biggest challenge even in the perspective of direct contact.

Nature-based interventions in virtual reality following this model would have been useful during the COVID-19 pandemic that led to a global lockdown to contain the spread of the virus and “flatten the curve” of the pandemic, which resulted in far-reaching effects in different strata of life including the increase in mental health, well-being, and quality of life challenges, among others (Onyeaka et al., 2021). Nature-based 2D and 3D experiences with forests, beach and water were helpful in managing stress during the pandemic (Beverly et al., 2022; Riches et al., 2023). This situation made people to reflect on how much direct or even indirect contact with nature could be useful for mental health. It is possible to infer that in the absence of urgent measures to mitigate climate change, the likelihood of future pandemics may increase. However, by reflecting on the lessons learned from the current pandemic, society can better prepare to confront such challenges. Nature-based interventions can play a crucial role in this preparation, enabling effective responses and mitigating the impact of future pandemics. Recognizing the importance of nature-based interventions bolsters resilience and fosters well-being in the face of adversity. These interventions harness the healing power of nature, offering a valuable tool to support individuals and communities in times of crisis.

Currently, there are some virtual reality simulators and apps that allow users to explore and interact with beautiful natural landscapes, including forests, beaches, and mountains. Others can offer an immersive experience in the underwater world, allowing users to interact with different marine species and ecosystems. Submarines or virtual encounters with wild animals in their natural habitat. These technologies can also produce well-being (Frewen et al., 2020; Gerçeker et al., 2020; Adhyaru and Kemp, 2022). Although such experiences can help in the levels of connection with nature, their reach both for human health and for the conservation of biodiversity are still not well established and also were not built considering the four proposed levels of experiences. New applications can be developed in the light of this model so that educational and active experiences are incorporated, which enables the expected results that it proposes to achieve. It is noteworthy that the current model needs to be tested to validate its effectiveness in the real world. To this end, a randomized controlled clinical trial is underway that has already included more than 400 participants in five natural units (urban, peri-urban, and rural) (ClinicalTrials.gov ID NCT05486156). This clinical trial aims to assess the mean change in the World Health Organization Wellness Index, changes in self-perceived happiness, vitality, connectedness, and engagement with nature at pre-intervention, post-intervention, and 30-day follow-up moments. In the intervention group (based on the proposed model) in addition to including walking in nature with attention to the senses (which was carried out by the control group) the researchers associate other elements of a cognitive-behavioral nature, aiming at human and non-human well-being. Thus, in this nature-based health intervention, activities related to four types of experience are proposed (1- Aesthetic and Emotional; 2- Multisensory integration; 3 - Knowledge; 4- Engagement) according to the activities proposed in this model.

Understanding the relationship of interdependence between all beings, facilitated by the four levels of experience of the proposed model, from corresponding activities can configure a relationship between human beings and nature in the future in the medium and long term.

## Data availability statement

The original contributions presented in the study are included in the article/supplementary material, further inquiries can be directed to the corresponding author.

## Author contributions

EL wrote the first draft of the manuscript. EL, EH-Z, and RS contributed to conception and design of the study. KP, LL, and GB revised the manuscript critically. All authors wrote sections of the manuscript and approved the submitted version.

## Funding

This study was supported by the Boticário Group Foundation for Nature Protection.

## Acknowledgments

We thank Luciana Cavalheiro Marti, researcher at the Albert Einstein Education and Research Center who kindly helped us to improve the figures contained in the article.

## Conflict of interest

The authors declare that the research was conducted in the absence of any commercial or financial relationships that could be construed as a potential conflict of interest.

## Publisher’s note

All claims expressed in this article are solely those of the authors and do not necessarily represent those of their affiliated organizations, or those of the publisher, the editors and the reviewers. Any product that may be evaluated in this article, or claim that may be made by its manufacturer, is not guaranteed or endorsed by the publisher.

## Footnotes

^1^This material is available upon request from the corresponding author.
